# Study on the Occurrence of Genetic Exchange Among Parasites of the *Leishmania mexicana* Complex

**DOI:** 10.3389/fcimb.2020.607253

**Published:** 2020-12-07

**Authors:** Roman Telittchenko, Albert Descoteaux

**Affiliations:** Institut national de la recherche scientifique, Centre Armand-Frappier Santé Biotechnologie, Laval, QC, Canada

**Keywords:** genetic exchange, *Leishmania*, host-pathogen relationship, macrophage, drug resistance, intracellular pathogen

## Abstract

In *Leishmania*, genetic exchange has been experimentally demonstrated to occur in the sand fly vector and in promastigote axenic cultures through a meiotic-like process. No evidence of genetic exchange in mammalian hosts have been reported so far, possibly due to the fact that the *Leishmania* species used in previous studies replicate within individual parasitophorous vacuoles. In the present work, we explored the possibility that residing in communal vacuoles may provide conditions favorable for genetic exchange for *L. mexicana* and *L. amazonensis*. Using promastigote lines of both species harboring integrated or episomal drug-resistance markers, we assessed whether genetic exchange can occur in axenic cultures, in infected macrophages as well as in infected mice. We obtained evidence of genetic exchange for *L. amazonensis* in both axenic promastigote cultures and infected macrophages. However, the resulting products of those putative genetic events were unstable as they did not sustain growth in subsequent sub-cultures, precluding further characterization.

## Introduction

Protozoan parasites of the genus *Leishmania* are the causative agents of a spectrum of diseases known as leishmaniasis that range from self-healing cutaneous lesions to destructive mucocutaneous infections and visceral pathologies. *Leishmania* has a distinct life cycle which consists of two specific environments. The first is that of the sand fly insect vector in which the parasites multiply within the alimentary tract under the promastigote form and the second is the infected mammalian or human hosts where the parasites replicate as amastigotes within the phagolysosomal compartment of host phagocytes. Currently, there are 20 known species of parasites that are associated with human disease. However, there is still a considerable amount of debate of whether this diversity is due to recombinational events or due to gradual accumulation of mutations during clonal division (Tibayrenc and Ayala, 2013; Rougeron et al., 2017).

In eukaryotic pathogenic organisms, sex is one of the main mechanisms that allows the spread of pathogenicity, resistance, and virulence genes (Heitman, 2010). Due to very strong linkage disequilibrium observed in *Leishmania*, it has been argued that the reproductive mode of *Leishmania* is predominantly clonal (Tibayrenc and Ayala, 2013). However, there is much evidence indicating that genetic exchange is part of the biology of *Leishmania* parasites, as evidenced by the occurrence of hybrids in nature. These natural hybrids were described at the intraspecific level for *L. tropica, L. donovani, L. infantum*, and *L. brasiliensis* (Chargui et al., 2009; Rougeron et al., 2009; Gelanew et al., 2014; Rogers et al., 2014; Iantorno et al., 2017). There were also reports of hybrids that originated from crosses between parasites of the *Viannia* subgenus, such as *L. braziliensis* and *L. guyanensis*, which are one of the most common ones described (Bonfante-Garrido et al., 1992; Belli et al., 1994; Dujardin et al., 1995; Banuls et al., 1997; Cupolillo et al., 1997; Delgado et al., 1997; Banuls et al., 1999; Torrico et al., 1999; Nolder et al., 2007; Cortes et al., 2012; Jennings et al., 2014; Kato et al., 2016; Kato et al., 2019). Natural hybrids were also reported for *Leishmania* species of the *Leishmania* subgenus such as *L. major* and *L. arabica*, *L. major* and *L. infantum*, as well as *L. donovani* and *L. infantum* (Evans et al., 1987; Kelly et al., 1991; Ravel et al., 2006; Volf et al., 2007; Odiwuor et al., 2011; Seblova et al., 2015; Cortes et al., 2019).

Using two strains of *L. major* harboring distinct integrated drug-resistance markers, Akopyants and colleagues experimentally demonstrated the existence of genetic exchange in the invertebrate stage of the parasite (Akopyants et al., 2009). By infecting sand flies and dissecting them 13–16 days post-infection, the double drug-resistant progeny of this cross was further demonstrated to be actual genomic hybrids by confirming the presence of at least one set of allelic markers from each parent (Akopyants et al., 2009). In another study from the same group, it was further shown that crosses in the invertebrate stage between *L. major* parasites coming from 4 distinct geographical locations are able to produce hybrid progeny, which also suggests that there are no intraspecies barriers when it comes to exchanging genetic information (Inbar et al., 2013). Another interesting finding, was that hybrid formation was observed in both the natural *P. duboscqi* vector and in the unnatural but permissive *L. longipalpis* and, by isolating the parasites from infected sand flies 3–18 days post-infection, it was further ruled out that genetic exchange takes place between parasites when they are in the nectomonad form (Inbar et al., 2013). In addition, a study based on microscopy and flow cytometry allowed to visualize evidence of genetic exchange between two strains of *L. donovani* expressing two different fluorescent molecules (RFP and GFP) which were present in the same vector (*P. perniciosus* or *L. longipalpis*) and gave rise to yellow promastigote progeny; however, these putative hybrids could not be recovered from the sand flies and grown in culture for further analyses (Sadlova et al., 2011). There was also a study which demonstrated hybrid formation in sand flies between two *L. infantum* strains expressing different fluorescent as well as different drug-resistance markers (Calvo-Alvarez et al., 2014) and another paper demonstrated formation of hybrid parasite strains in sand flies between two entirely different species, namely *L. major* and *L. infantum* (Romano et al., 2014). Finally, the ability of *L. tropica* to exchange genetic information in an intraspecific manner in an infected insect vector as well as in axenic culture has also been recently demonstrated using whole genome sequencing (Inbar et al., 2019; Louradour et al., 2020).

Despite the fact that hybrid parasites could be isolated both in nature and in laboratory conditions from infected sand flies and axenic cultures, the mechanism by which they reproduce is still poorly understood. This is partially due to the fact that this is not an obligate mode of reproduction of the parasite; however, recent genome sequencing data from 44 hybrids generated between and within *L. infantum*, *L. tropica*, and *L. major* suggest that *Leishmania* reproduces *via* a meotic-like mechanism (Inbar et al., 2019). Apart from one study using *L. major* (Akopyants et al., 2009), it is still not widely known whether or not genetic exchange can occur within an infected mammalian host, although there is a study that has shown previously by DNA quantification that infected macrophages could harbor 4N amastigotes suggesting that genetic exchange is possible in mammalian host cells (Kreutzer et al., 1994). Here, we explored the possibility of intraclonal and interspecific genetic exchange among parasites of the *L. mexicana* complex, which unlike other *Leishmania* species, replicate in spacious communal vacuoles that may provide an environment favorable to genetic exchange (Case et al., 2016).

## Materials and Methods

### Ethics Statement

All animal handling was performed in accordance with the protocols 1806–01 and 1806–02, which were approved by the *Comité Institutionel de Protection des Animaux* of the INRS-Centre Armand-Frappier Santé Biotechnologie. These protocols respect procedures on animal practice as instructed by the Canadian Council on Animal Care, described in the Guide to the Care and Use of Experimental Animals.

### Plasmids and Constructs

**Figure 1:**
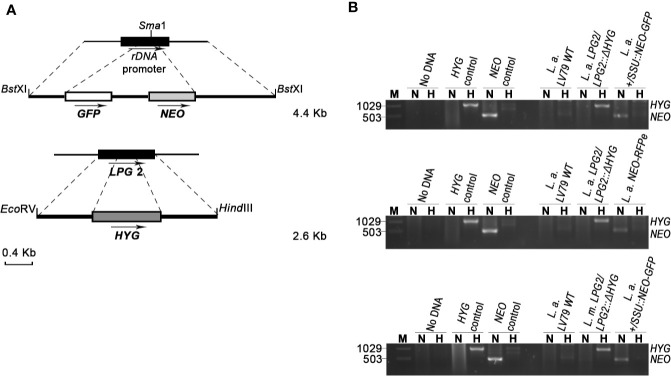
Generation of drug-resistant *Leishmania* parasites. **(A)** L.d-rDNA-*NEO-GFP* and *LPG2::ΔHYG* targeting constructs were used for the integration into the ribosomal RNA locus or in one allele of *LPG2*, respectively. For the L.d-rDNA-*NEO-GFP* construct, the *NEO-GFP* resistance cassette (white and gray boxes) was inserted in the *SmaI* site of the ribosomal RNA locus (black rectangle). The dashed lines delimit the regions of recombination between the target genes and targeting constructs. Arrows indicate orientation. **(B)** PCR products for drug resistance markers *HYG* and *NEO* of *L. amazonensis* and *L. mexicana* parental strains. The size of *HYG* and *NEO* resistance genes is 1,029 and 503 bp long, respectively. The pLeish-*HYG*-*GFP* and the pKS-*NEO-DsRed* constructs were used as controls for the *HYG* and *NEO* genes, respectively. *L.a.* LV79 WT is a DNA sample used to show that our wild type parasites do not harbor any drug-resistance markers in their genomes. *L. amazonensis LPG2/LPG2::ΔHYG, L. amazonensis +/SSU::NEO-GFP, L. mexicana LPG2/LPG2::ΔHYG* and *L. amazonensis NEO*-*DsRed*e are controls used to validate the presence of *HYG* and *NEO* resistance genes. No DNA sample was loaded as negative control. M, molecular DNA ladder; H, Hygromycin; N, G418.

The plasmid pLa*LPG2*-*HYG* from which the *LPG2::ΔHYG* targeting construct was used to create Hygromycin B-resistant parasites was kindly provided by Drs. Valeria M. Borges and Leonardo Paiva Farias (Fiocruz Bahia - Instituto Gonçalo Moniz, Brazil) (**Figure 1**). The plasmid pCR2.1-L.d-rDNA-pr-αIR*NEO*αIR-*GFP* from which the Ld-rDNA-*NEO-GFP* targeting sequence was used to create G418-resistant parasites was kindly provided by Dr. Barbara Papadopoulou (Université Laval, Canada) (**Figure 1**). The plasmid pKS-*NEO-DsRed* was provided by Dr. David L. Sacks (National Institute of Allergy and Infectious Diseases, USA) (Kimblin et al., 2008). The pLeish-*HYG*-*GFP* construct was created the following way: a *Sac*I fragment containing the *GFP* gene was excised from the plasmid pXG-*GFP*^+^ (Ha et al., 1996), blunted, and inserted into the *EcoR*V site of pLeish-*HYG* (unpublished), yielding pLeish-*HYG-GFP*.

### Parasites

Both *L. amazonensis* LV79 (MPRO/BR/72/M1841) and *L. mexicana* (MNYC/BZ/62/M379) were passaged in mice to maintain their virulence. Amastigotes recovered from ear dermis lesions of infected C57BL/6 mice were differentiated into promastigotes in *Leishmania* medium (M199-1X (Sigma) with 10% heat-inactivated fetal bovine serum (FBS), 100 μM hypoxanthine, 3 μM biopterin, 40 mM HEPES at pH 7.4, 5 μM hemin, 1 μM biotin, and Penicillin-Streptomycin) in a 26°C incubator. For the generation of *L. amazonensis LPG2/LPG2::ΔHYG* and *L. mexicana LPG2/LPG2::ΔHYG*, log-phase *L. amazonensis* and *L. mexicana* promastigotes were electroporated with the *LPG2::ΔHYG* targeting construct (excised as a 2.6-kb *EcoRV*I-*Hind*III-*Bgl*I fragment from pLa*LPG2*KO-*HYG*) in 0.2 cm electroporation cuvettes, at 0.45 kV and 500 µF of high capacitance as previously described in similar protocols (Descoteaux et al., 1994; Turco et al., 1994). After electroporation, promastigotes were grown in drug-free *Leishmania* medium for 24 h. Following this incubation, *L. amazonensis LPG2/LPG2::ΔHYG* parasites were selected in the presence of 35 µg/ml Hygromycin B (Sigma) and *L. mexicana LPG2/LPG2::ΔHYG* parasites were selected in the presence of 70 µg/ml Hygromycin B (Sigma) respectively. For the generation of *L. amazonensis* +/*SSU::NEO-GFP*, *L. amazonensis* promastigotes were electroporated with the L.d-rDNA-*NEO-GFP* targeting construct (excised as a 4.25-kb *Bst*XI fragment from pCR2.1-L.d-rDNA-pr-αIR*NEO*αIR-*GFP*). After electroporation, the parasites were grown in drug free medium for 24 h and then grown in *Leishmania* medium containing 20 µg/ml G418 (Life Technologies). *L. amazonensis NEO-DsRed*e parasites were obtained by electroporating *L. amazonensis* promastigotes with the plasmid pKS-*NEO-DsRed*. Parasites were grown in drug free medium for 24 h and then grown in medium containing 20 µg/ml G418. The same method was used to obtain *L. mexicana* pKS-*NEO-DsRed*e and they were maintained in *Leishmania* medium containing 40 µg/ml of G418. *L. amazonensis HYG-GFP*e promastigotes were generated by electroporating *L. amazonensis* with the plasmid pLeish-*HYG-GFP*. Parasites were grown in drug-free medium for 24 h and then grown in medium containing 35 µg/ml Hygromycin B

### Mammalian Cell Culture

Bone marrow-derived macrophages (BMM) were differentiated from the bone marrow of 6- to 8-week old C57BL/6 mice as previously described (Descoteaux and Matlashewski, 1989). BMM were differentiated for 7 days in complete DMEM [containing L-glutamine (Life Technologies), 10% v/v heat inactivated fetal bovine serum (FBS) (Life Technologies), 10 mM HEPES (Bioshop) at pH 7.4, and penicillin-streptomycin (Life Technologies)] supplemented with 15% v/v L929 cell-conditioned medium (LCM) as a source of macrophage colony-stimulating factor-1. To render the BMM quiescent prior to experiments, cells were transferred to tissue culture-treated 6- or 24-well plates or T25 tissue culture flasks for 24 h in complete DMEM without LCM. The cells were kept in a humidified 37°C incubator with 5% CO_2_. The number of macrophages used per container are as following: 2.2 X 10^6^ BMMs per well of a 6-well plate, 0.3 X 10^6^ BMMs per well of 24-well plate and 25 X 10^6^ BMMs in T-25 flasks.

### Transwell Experiments

For genetic exchange transwell experiments, donor parasites (*L. amazonensis NEO-dsRed*e) were relocated to the insert chamber containing 0.4 µm pores in a polycarbonate membrane (Corning) and the recipient parasites (*L. amazonensis LPG2/LPG2::ΔHYG*) were added to the wells. The plates were then either incubated at 26°C or pre-incubated at 34°C for 4 h, as done previously (Hassani et al., 2011), and then transferred to 26°C. The parasites were collected at 24, 72, 96, and 120 h post-incubation. Each parental stain was equally divided into 3 wells of a 6-well plate and were grown in the presence of antibiotics. Two wells were used as controls containing either 35 µg/ml of Hygromycin B or 20 µg/ml of G418 and the last well contained both drugs in the medium. The parasites were kept in such conditions up to 3 weeks. Each parental strain was also grown separately and were under the same conditions as a control.

### Parasite Co-Culture Experiments

As described (Louradour et al., 2020), stationary phase promastigotes of two parental strains were mixed and distributed into 96-well plates up to a total volume of 100 µl in each well. One million parasites of each strain were added in the wells. Three days later, each co-culture from the 96-well plate was transferred to a single well of a 24-well plate containing 900 µl of *Leishmania* medium containing either 35 µg/ml Hygromycin B and 20 µg/ml G418 if both parental strains were *L. amazonensis* or 60 µg/ml Hygromycin B and 40 µg/ml G418 if one of the parental strains was *L. amazonensis* and the other was *L. mexicana*. Each line was cultured individually in *Leishmania* medium supplemented with either Hygromycin B or G418 or both drugs as controls. When double drug-resistant parasite cultures were growing in wells (growth was observed between 19 and 28 days), the cells were passaged in *Leishmania* medium at a dilution of 1:10. DNA was then extracted from double drug-resistant parasites and was used for PCR reactions.

### *In Vitro* Infections

Metacyclic promastigotes were isolated from promastigote cultures in the late stationary phase by means of a density gradient centrifugation (Spath and Beverley, 2001). Specifically, 2 ml of 40% w/v Ficoll PM400 (GE healthcare) were added to the bottom of ta 15 ml tube, followed by a 5 ml layer of 10% Ficoll PM400 in M199-1x and topped by late stationary phase promastigotes resuspended in 5 ml of DMEM with no FBS (Arango Duque et al., 2019). Metacyclic promastigotes were collected from the DMEM-10% Ficoll interphase after spinning the gradient for 10 min. The percentage of isolated metacyclic parasites from the interphase generally varied from 12–18% of the input population. Metacyclic promastigotes were then opsonized with the serum of C57BL/6 mice for 30 min, washed 3 times with PBS and resuspended in cold complete DMEM (cDMEM). The parasites were then fed to macrophages adhered in T-25 flasks (Sarstedt) (Ratio 3:1 for single infections, ratio 6:1 for mix infections). The cells were then incubated at 4°C for 10 min (Arango Duque et al., 2019) to synchronize phagocytosis. The internalization of parasites was triggered by transferring the cells to 34°C (Arango Duque et al., 2019). Two hours post-internalization, the cells were washed three times with warmed cDMEM to remove non-internalized promastigotes. Infected BMM were incubated for 120 and 192 h. Next, the amastigotes were isolated from infected macrophages by resuspending those in cDMEM containing 0.05% of SDS. Shortly, the macrophages resuspended in 2ml of cDMEM containing SDS are incubated at 37°C for 3 min. Then, the resulting supernatant is resuspended in 10 ml of cDMEM and spun at 3,000 rpm. After the spin, the supernatant was discarded. The amastigotes were resuspended in *Leishmania* medium and separated into 3 separate conditions. The conditions were: *Leishmania* medium containing 20 µg/ml of G418 or *Leishmania* medium containing 32 µg/ml of Hygromycin B or *Leishmania* medium containing both drugs. The parasites were left for incubation at 26°C for up to 3 weeks to select for double drug-resistant parasites. If applicable, the double drug-resistant parasites were passaged at a dilution of 1/10 and their DNA was then extracted and was used for PCR reactions. Double drug-resistant parasites were also passaged in infected BMM for 3 days as well. For parasite survival, cells were washed with PBS and fixed and stained with fixative and staining solutions of the Hema 3 stain set (Fisher Scientific). This process was done for 2, 48, 120, and 192 h timepoints.

Alternatively, the infections were done in 6-well plates instead of T-25 flasks. Three wells were used for mixed infection for each timepoint (120 and 192 h) and two wells were reserved for infection with each parental strain alone. Once the amastigotes were obtained, they were plated in 96-well plates in 100 µl of drug free *Leishmania* medium as described in the parasite co-culture section. The amastigotes were plated at 5 million parasites per well. Three days later, each well was transferred to a well of 24-well plate that contained 900 µl with antibiotics. Pure parental cultures were used as controls as previously described. If applicable, the double drug-resistant parasites were passaged at a dilution of 1/10 and their DNA was then extracted from double drug-resistant parasites and was used for PCR reactions.

### *In Vivo* Infections and Parasite Recovery

C57BL/6 mice (6–8 weeks old) were infected with 1 X 10^5^ metacyclic promastigotes (5 X 10^4^ of each line) of either *L. amazonensis LPG2/LPG2::ΔHYG* + *L. amaz* +/*SSU::NEO-GFP* or *L. mexicana LPG2/LPG2::ΔHYG* + *L. amaz* +/*SSU::NEO-GFP* into the ear dermis with an insulin syringe (29 G). Mice infected separately with each line were used as controls. At 9 weeks post-infection, mice were euthanized under CO_2_ asphyxiation and by cerebral dislocation as well. The infected ears were then collected and disinfected in 70% ethanol for 10 min and air dried for 10 min. Then, they were separated into dorsal and ventral leaflets and cut up into small pieces with surgical scissors. The cut-up ears were loaded in 2.0 ml tubes containing zirconium beads (Benchmark Scientific Inc.) and resuspended in 1 ml of *Leishmania* medium and vortexed for a 1 min and 30 s. The resulting suspension was then transferred to 100 µm cell strainers placed over 50 ml Falcon tubes and filtered to isolate the amastigotes. The remaining tissue in the cell strainer was smashed with a sterile 10 ml syringe plunger and washed two times with *Leishmania* medium. The resulting cell suspension was spun at 3,200 RPM at 4°C for 10 min. The amastigotes were then separated in three T-25 flasks and left in unconditioned *Leishmania* medium for 24 h. Lastly, the antibiotics were added to each flask according to each condition and were incubated at 26°C for three weeks. The conditions were Hygromycin only, G418 only or both drugs.

### DNA Extraction and PCR Confirmation of Double-Resistant Parasites

For genotyping analyses, total DNA was extracted from parasites by using a phenol/chloroform treatment as previously described (Medina-Acosta and Cross, 1993). All of the PCR amplifications were done in 50 µl total volume containing 100 ng of parasite DNA and 10pmol of each primer. The following primer pairs were used: for Hygromycin B 5’-ATGAAAAGCCTGAACTCACC-3’ (Forward), 5’-CTATTCCTTTGCCCTCGG-3’ (Reverse) that were previously described (Romano, 2014); for G418 5’CCACGACGGGCGTTCCTTGCGCAGCTGTGC-3’ (Forward), 5’-GTCAGCCCATTCG CCAAGCTCTTCAGC-3’ (Reverse) which were custom made. The resulting DNA products were then verified by electrophoresis on 1.2% Agarose gel and subsequently viewed by staining the samples with ethidium bromide.

### Live Microscopy

BMMs were platted at the bottom of 6 well-plate with a coverslip attached to the bottom of the wells. The cells were kept in the 34°C incubator for 24 h without LCM to render them quiescent. They were then infected with metacyclic parasites of each line separately as a positive control or with a combination of two. Non-infected cells were used as a negative control. The samples were then viewed with 63X objective lens LSM780 system confocal microscope (Carl Zeiss microimaging). The images were taken and processed with the ZEN 2012 Software (Carl Zeiss) and subsequently mounted into the figures *via* Adobe Photoshop 2019.

## Results

### Generation of Drug-Resistant Strains of *L. amazonensis* and *L. mexicana*

**Figure 2:**
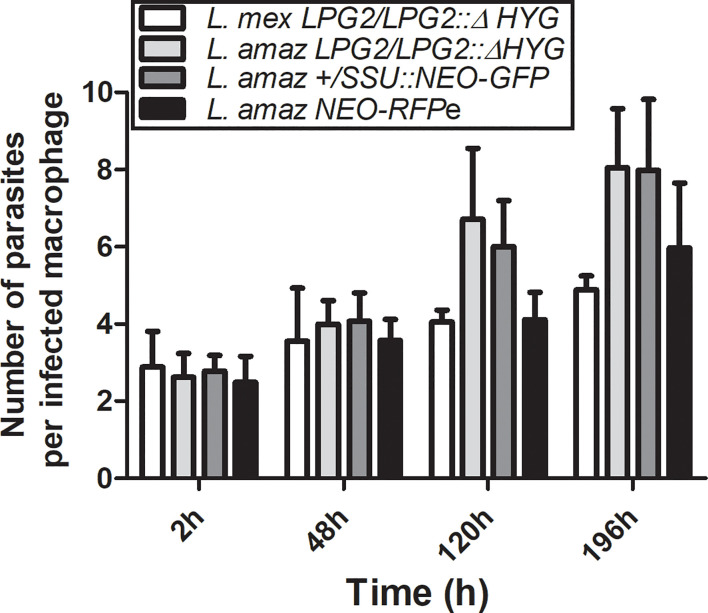
Survival of parental strains within infected macrophages. BMMs were infected with metacyclic serum-opsonized promastigotes of *L. amazonensis* and *L. mexicana* parental strains (*L. amazonensis LPG2/LPG2::ΔHYG*, *L. mexicana LPG2/LPG2::ΔHYG, L. amazonensis +/SSU::NEO-GFP, L. amazonensis NEO*-*DsRed*e) for 2, 48, 120, and 196 h. Bars represent mean ± SE of three representative experiments performed in triplicate in bone marrow derived murine macrophages. Parasites were counted in 100 macrophages and quantified by light microscopy.

To investigate the possibility that formation of hybrids and genetic exchange may occur among parasites of the *L. mexicana* complex, we used *L. amazonensis* LV79 and *L. mexicana* M379 expressing either episomal or integrated genes encoding resistance to Hygromycin B (*HYG*) or to G418 (*NEO)*. To this end, we generated one line of *L. amazonensis* and one line of *L. mexicana* in which the *HYG* resistance gene was integrated in one allele of the *LPG2* gene (*L. amazonensis LPG2/LPG2::ΔHYG* and *L. mexicana LPG2/LPG2::ΔHYG*) (**Figure 1A**), one line of *L. amazonensis* in which a *NEO*-*GFP* construct was integrated into the ribosomal RNA locus (*L. amazonensis +/SSU::NEO-GFP*) (**Figure 1A**), one line of *L. amazonensis* and one line of *L. mexicana* with an episomal *NEO-DsRed* plasmid (*L. amazonensis NEO*-*DsRed*e and *L. mexicana NEO-DsRed*e), and one line of *L. amazonensis* with an episomal *HYG-GFP* plasmid (*L. amazonensis HYG-GFP*e). We confirmed the presence/absence of both resistance genes in each line by PCR analysis using specific primers against *HYG* and *NEO* (**Figure 1B**), and we ensured that these drug-resistant recombinant parasites retained the ability to infect and replicate within bone marrow-derived macrophages (BMMs) over a period of 196 h (**Figure 2**).

### Drug Resistance is not Transferred in *In Vitro* Cultures of Promastigotes in the Absence of Cell-To-Cell Contact

Evidence indicate that DNA can be transferred from cell-to-cell through extracellular vesicles (Elzanowska et al., 2020). In addition, erythrocytes infected with *Plasmodium falciparum* can transfer parasite DNA to other infected cells *via* the release of extracellular vesicles (Regev-Rudzki et al., 2013). Whereas no such mechanism has been described in *Leishmania*, it was recently reported that the *Leishmania* RNA virus 1 (LRV1) exploits the *Leishmania* exosomal pathway as a mode of transmission from one promastigote to another (Atayde et al., 2019). This led us to verify the hypothesis that extracellular vesicles released in the culture medium may serve as a vehicle to transfer genetic material, including episomes harboring a drug-resistance gene among promastigotes. To this end, we used transwells (0.4 µm pores) to physically separate *L. amazonensis NEO-DsRed*e promastigotes from *L. amazonensis*-*LPG2/LPG2::ΔHYG* promastigotes. We incubated the transwell plates either at 26°C or we pre-incubated them at 34°C for 4 h and then transferred the plates to 26°C. Such a transient increase in temperature has been previously shown to enhance the secretion of extracellular vesicles by *Leishmania* promastigotes (Hassani et al., 2011). Promastigotes co-incubated in transwells at either 26 or 34°C were collected after 24, 72, 96, and 120 h and assessed for their capacity to grow in the presence of both hygromycin and G418. Both *L. amazonensis*-*NEO*-*DsRed*e and *L. amazonensis*-*LPG2/LPG2::ΔHYG* were viable and resistant to G418 and hygromycin, respectively, up to 120 h of co-incubation in the transwells. However, no double drug-resistant parasites were recovered from 9 independent experiments performed in triplicate, indicating that exchange of genetic information through extracellular vesicles among *L. amazonensis* promastigotes, if it occurs, is a very rare event.

### Genetic Exchange Among *L. amazonensis* and *L. mexicana* Promastigotes in Axenic Cultures

**Figure 3:**
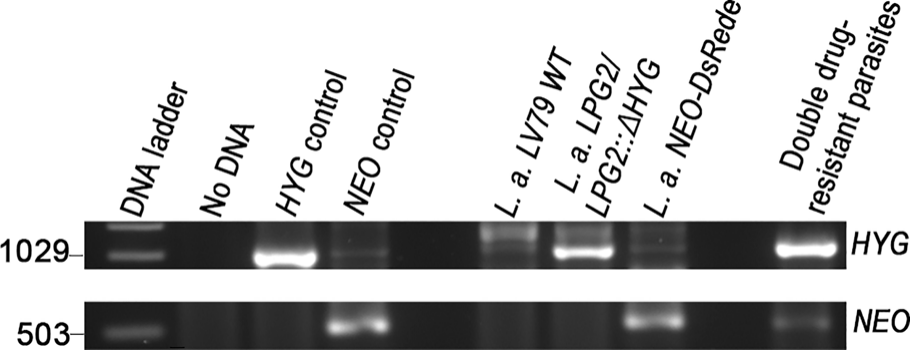
Molecular genotype characterization of double drug-resistant parasites isolated from axenic cultures. PCR amplification of genes encoding antibiotic resistance. The size of *HYG* and *NEO* resistance genes is 1,029 and 503 bp long. The pLeish-*HYG*-*GFP* and the pKS-*NEO-DsRed* constructs were used as controls for the *HYG* and *NEO* genes, respectively. *L.a.* LV79 WT is a DNA sample used to show that our wild type parasites do not express any drug-resistance markers. *L. amazonensis LPG2/LPG2::ΔHYG* and *L. amazonensis NEO*-*DsRed*e are controls used to validate the presence of *HYG* and *NEO* resistance genes within the appropriate parental strains. No DNA sample was loaded as negative control.

A recent study revealed that some strains of *L. tropica*, but not *L. major*, form hybrids in promastigote axenic cultures (Louradour et al., 2020). This finding prompted us to evaluate the occurrence of genetic crosses among *L. amazonensis* and *L. mexicana* promastigotes in *in vitro* co-cultures. Following the experimental protocol described by Louradour et al. we co-cultured combinations of stationary phase promastigotes with integrated drug-resistance genes as depicted in **Table 1** (Louradour et al., 2020). We also performed co-culture experiments using promastigotes harboring episomes (**Table 1**). Each co-culture was distributed into 96-well plates in drug-free medium. Three days later, the parasites were transferred into 24-well plates and cultured in selective medium (hygromycin B and G418) and left for up to 40 days in a 26°C incubator. Individual single drug-resistant lines were used as controls and had gone through the same process. After 40 days of incubation, we did not obtain double drug-resistant parasites except for the co-cultures of *L. amazonensis*-*LPG2/LPG2::ΔHYG* and *L. amazonensis-NEO*-*DsRed*e (**Table 1**). We obtained promastigote populations resistant to both G418 and hygromycin B in 3 separate wells. However, only one out of 3 grew sufficiently to allow for DNA isolation and PCR analysis, which revealed the presence of both *HYG* and *NEO* genes (**Figure 3**). However, we were unable to further characterize these double-drug-resistant parasites as they perished in subsequent sub-cultures. These results suggest that genetic exchange in axenic cultures among those two drug-resistant lines may occur and results in transient/unstable double drug-resistant promastigotes.

**Table 1:** Crosses used in axenic cultures.

Crosses in axenic cultures	No. of wells with crosses	% Yield of double drug-resistant parasites
*L. amaz LPG2/LPG2::ΔHYG* × *L. amaz +/SSU::NEO-GFP*	192	0/192 (0%)
*L. amaz LPG2/LPG2::ΔHYG* × *L. amaz NEO-DsRed*e	192	3/192 (1.56%)
*L. mex. LPG2/LPG2::ΔHYG* × *L. amaz +/SSU::NEO-GFP*	96	0/96 (0%)
*L. mex LPG2/LPG2::ΔHYG* × *L. amaz NEO-DsRed*e	96	0/96 (0%)

Demonstrates the number of wells tested and percentage of isolated double drug-resistant parasites.

### Unstable Genetic Exchange in Infected Macrophages

**Figure 4:**
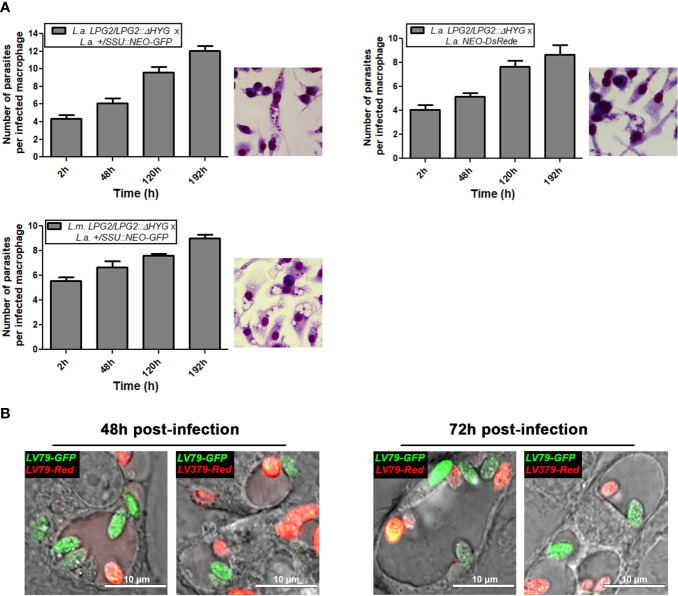
Survival of mating crosses within infected macrophages and visualization of both parental strains within the same vacuole. **(A)** BMMs were infected with metacyclic serum-opsonized promastigote crosses of *L. mexicana* complex parental parasite strains (*L. amazonensis LPG2/LPG2::ΔHYG* + *L. amazonensis +/SSU::NEO-GFP*; *L. amazonensis LPG2/LPG2::ΔHYG* + *L. amazonensis NEO*-*DsRed*e; *L. mexicana LPG2/LPG2::ΔHYG* + *L. amazonensis +/SSU::NEO-GFP* for 2, 48, 120, and 196 h. Bars represent mean ± SE of three representative experiments performed in triplicate in bone marrow derived murine macrophages. Parasites were counted in 100 macrophages and quantified by light microscopy. Macrophages were stained with HEMA 3 kit. Representative pictures from each cross are shown. **(B)** Live microscopy analysis of *L. amazonensis* and *L. mexicana* parasite strains expressing different fluorescent markers. Representative pictures of both parental strains within the same communal vacuole at 48 and 72 h are shown. *LV79-GFP*, *L. amazonensis HYG-GFPe*; *LV79-DsRed*, *L. amazonensis NEO-DsRed*e; *M379-DsRed*, *L. mexicana NEO-DsRed*e.

**Figure 5:**
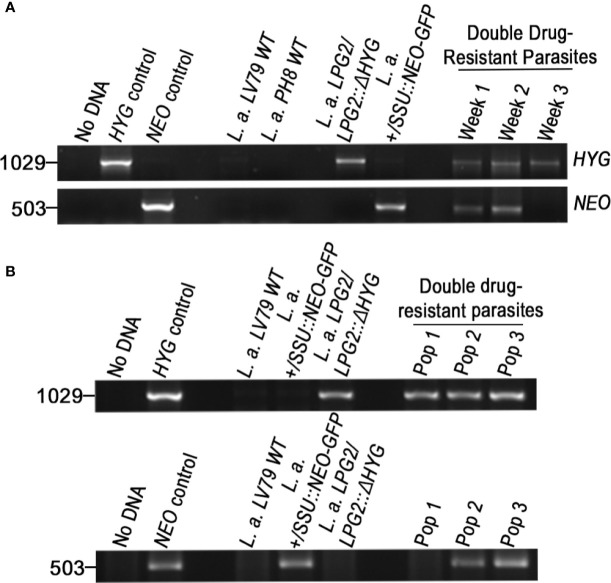
Molecular genotype characterization of double drug-resistant parasites isolated from *in vitro* infections. PCR amplification of genes encoding antibiotic resistance. The size of *HYG* and *NEO* resistance genes is 1,029 and 503 bp long. The pLeish-*HYG*-*GFP* and the pKS-*NEO-DsRed* constructs were used as controls for the *HYG* and *NEO* genes, respectively. *L.a.* LV79 WT and *L.a.* PH8 WT is a DNA sample used to show that our wild type parasites do not express any drug-resistance markers. *L. amazonensis LPG2/LPG2::ΔHYG, L. amazonensis +/SSU::NEO-GFP* are controls used to validate the expression of *HYG* and *NEO* resistance genes within the appropriate parental strains. No DNA sample was loaded as negative control. **(A)** PCR amplification of resistance genes of the first double drug-resistant parasite population. Population was maintained for 3 weeks until it lost one of the resistance genes and perished. PCRs of double drug-resistant parasites represent the presence of both genes on weeks 1, 2, and 3 **(B)** PCR amplification of resistance genes of the second occurrence of double drug-resistant parasites. Three populations were isolated (Pop 1–3). Two of the population were found to be double-drug resistant and one was not.

The fact that *L. amazonensis* and *L. mexicana* replicate within communal parasitophorous vacuoles led us to verify the possibility that these intracellular replicative niches provide conditions propitious for genetic exchange. To this end, we infected BMMs with the following four combinations of drug-resistant parasites: *L. amazonensis LPG2/LPG2::ΔHYG* + *L. amazonensis +/SSU::NEO-GFP*; *L. amazonensis LPG2/LPG2::ΔHYG* + *L. amazonensis NEO*-*DsRed*e; *L. mexicana LPG2/LPG2::ΔHYG* + *L. amazonensis +/SSU::NEO-GFP*, and *L. mexicana LPG2/LPG2::ΔHYG* + *L. amazonensis NEO*-*DsRed*e. Similar to single infection, parasites in mixed infections replicated up to 192 h post-infection and induced the formation of communal PVs (**Figure 4A**). To confirm that these communal PVs harbored both drug-resistant *Leishmania* lines, we performed live cell imaging on BMMs co-infected with either *L. amazonensis HYG*-*GFP*e + *L. amazonensis NEO*-*DsRed*e or *L. amazonensis HYG*-*GFP*e + *L. mexicana NEO*-*DsRed*e. In both cases, we observed the two drug-resistant parasite lines within the same communal vacuoles, at 48 and 72 h post-infection (**Figure 4B**). At 120 and 192 h post-infection, we lysed the infected BMM and cultured the recovered parasites in medium containing hygromycin B and G418 in a similar fashion as the axenic parasite cultures done in plates. As shown in **Table 2**, we failed to recover any double drug-resistant parasites from these co-infection experiments. Next, we modified our experimental approach to perform co-infection experiments on a larger scale, with the following 3 combinations of drug-resistant promastigotes: *L. amazonensis LPG2/LPG2::ΔHYG* + *L. amazonensis +/SSU::NEO-GFP*, *L. amazonensis LPG2/LPG2::ΔHYG* + *L. amazonensis NEO*-*DsRed*e, and *L. mexicana LPG2/LPG2::ΔHYG* + *L. amazonensis +/SSU::NEO-GFP* (**Table 3**). We co-infected BMMs with each combination and we used each individual drug-resistant line as controls. Out of a total of 28 infections, we obtained double drug-resistant parasite populations out of 2 separate infections, which arose from the co-infections with *L. amazonensis LPG2/LPG2::ΔHYG* + *L. amazonensis +/SSU::NEO-GFP* (**Table 3**). We detected the presence of both *NEO* and *HYG* drug-resistance genes in these double drug-resistant parasite populations by PCR analysis (**Figure 5**). We were able to maintain one of this double drug-resistant population in culture for 3 weeks; however, after the third week, this population lost the *NEO* resistance gene and has ultimately perished afterwards (**Figure 5A**). For the second occurrence of double drug-resistant parasites, we isolated 3 separate populations which contained both the *HYG* and *NEO* genes as assessed by PCR analysis (**Figure 5B**), whereas the third population had only the *HYG* resistance gene and died upon further passages (**Figure 5B**). The two double drug-resistant populations were maintained for a week and died upon additional passages. These results suggest that genetic exchange may take place in infected macrophages and result in transient/unstable double drug-resistant parasites.

**Table 2:** Crosses used in *in vitro* infections done in wells.

Crosses 120 h post-infection	No. of wells with crosses	% Yield of double drug-resistant parasites
*L. amaz LPG2/LPG2::ΔHYG* × *L. amaz +/SSU::NEO-GFP*	89	0/89 (0%)
*L. amaz LPG2/LPG2::ΔHYG* × *L. amaz NEO-DsRed*e	34	0/34 (0%)
*L. mex. LPG2/LPG2::ΔHYG* × *L. amaz +/SSU::NEO-GFP*	63	0/63 (0%)
*L. mex LPG2/LPG2::ΔHYG* × *L. amaz NEO-DsRed*e	63	0/63 (0%)
**Crosses** **192 h post-infection**	**No. of wells with crosses**	**% Yield of double drug-resistant parasites**
*L. amaz LPG2/LPG2::ΔHYG* × *L. amaz +/SSU::NEO-GFP*	72	0/72 (0%)
*L. amaz LPG2/LPG2::ΔHYG* × *L. amaz NEO-DsRed*e	72	0/72 (0%)
*L. mex. LPG2/LPG2::ΔHYG* × *L. amaz +/SSU::NEO-GFP*	69	0/69 (0%)
*L. mex LPG2/LPG2::ΔHYG* × *L. amaz NEO-DsRed*e	66	0/66 (0%)

Demonstrates the number of wells tested and percentage of isolated double drug-resistant parasites.

**Table 3:** Crosses used in *in vitro* infections done in flasks.

Cross	No. of infections	No. of times parent 1 was isolated	No. of times parent 2 was isolated	No. of times double drug-resistant parasites were isolated	% Recovery
*L. amaz LPG2/LPG2::ΔHYG* × *L. amaz +/SSU::NEO-GFP*	14	14	14	2	14%
*L. amaz LPG2/LPG2::ΔHYG* × *L. amaz NEO-DsRed*e	10	10	10	0	0%
*L. mex. LPG2/LPG2::ΔHYG* × *L. amaz +/SSU::NEO-GFP*	4	4	4	0	0%

Data includes the number of mating crosses executed in flasks and parasite strains that were isolated from each infection. Percentage indicates the total number of times that double-drug resistant parasites were isolated.

### Absence of Detectable Genetic Exchange in *In Vivo* Infections

To determine whether mammalian hosts provide an environment favorable for genetic exchange for the species of the *L. mexicana* complex, we inoculated mice into the ear dermis with two combinations of single drug-resistant parasites, namely *L. amazonensis LPG2/LPG2::ΔHYG* + *L. amazonensis +/SSU::NEO-GFP* and *L. mexicana LPG2/LPG2::ΔHYG* + *L. amazonensis +/SSU::NEO-GFP* (**Table 4**). Mice infected with single drug-resistant lines were used as a control. Nine weeks post-infection, we recovered parasites from lesions and we cultured them in the presence of either hygromycin B, G418, or both. As shown in **Table 4**, we recovered each single drug-resistant line that was co-inoculated or inoculated alone as controls. However, we did not succeed in isolating double drug-resistant parasites from cutaneous lesions, indicating that genetic exchange does not occur to a detectable level within the mammalian host for *Leishmania* species residing in communal parasitophorous vacuoles.

**Table 4:** Crosses used in *in vivo* infections.

Cross	No. of infected mice	No. of times parent 1 was isolated	No. of times parent 2 was isolated	No. of times double drug-resistant parasites were isolated	% Recovery
*L. amaz LPG2/LPG2::ΔHYG* × *L. amaz +/SSU::NEO-GFP*	13	13	13	0	0%
*L. mex. LPG2/LPG2::ΔHYG* × *L. amaz +/SSU::NEO-GFP*	6	6	6	0	0%

Data includes the number of infected mice with each cross and parasite strains that were isolated from each infection. Percentage indicates the total number of times that double drug-resistant parasites were isolated.

## Discussion

For decades, the occurrence of natural *Leishmania* hybrids has been described among clinical and field isolates, indicating that genetic exchange is part of the biology of these parasites. Experimental genetic crosses among *Leishmania* cells were initially reported to occur exclusively in the sand fly vector (Akopyants et al., 2009; Sadlova et al., 2011; Inbar et al., 2013; Calvo-Alvarez et al., 2014; Romano et al., 2014; Inbar et al., 2019). However, recent evidence revealed that experimental genetic crosses also occur in axenic promastigote cultures, indicating that mating competent forms are present in these populations (Louradour et al., 2020). The fact that studies on the experimental generation of hybrids have been performed with *Leishmania* species living in tight individual parasitophorous vacuoles may have precluded the detection of genetic exchange within mammalian host cells. In this study, we sought to determine whether genetic exchange occurs among species of the *L. mexicana* complex, which replicate within communal parasitophorous vacuoles. Using promastigotes expressing drug-selectable markers, we obtained evidence of intraclonal genetic exchange for *L. amazonensis* in both axenic promastigote cultures and infected macrophages. However, the resulting products of those genetic events were unstable as they did not sustain growth in subsequent sub-cultures.

The study of experimental genetic exchange in *Leishmania* consists in mixing strains carrying distinct drug-resistance markers and/or fluorescent markers integrated into their genomes and the subsequent selection and analysis of double drug-resistant parasites (Akopyants et al., 2009; Sadlova et al., 2011; Inbar et al., 2013; Calvo-Alvarez et al., 2014; Romano et al., 2014; Inbar et al., 2019; Louradour et al., 2020). Whole genome sequencing revealed that these double drug-resistant parasites are full genomic hybrids predominantly resulting from a mechanism resembling meiosis (Inbar et al., 2019). Whether other forms of genetic exchange take place in *Leishmania* had not received much attention. Hence, we tested whether the transfer of genetic material can occur without direct contact between *Leishmania* promastigotes, as previously reported for *P. falciparum via* cell-derived extravesicular vesicles (Regev-Rudzki et al., 2013). Our attempts to detect the transfer of an episome from one line of *L. amazonensis* to another in transwell experiments were unsuccessful, suggesting that physical contact is required for genetic exchange among *Leishmania* promastigotes. Our results also suggest that in contrast to the *Leishmania* virus LRV-1 (Atayde et al., 2019), episomal DNA is not transferred through extracellular vesicles or other released material.

The recent report that genetic crosses take place in axenic cultures of *L. tropica* (Louradour et al., 2020) prompted us to explore the possibility that genetic exchange occur among *L. amazonensis* and *L. mexicana* promastigotes in axenic cultures. In contrast to the *L. tropica* strains used by Louradour and colleagues (Louradour et al., 2020), we obtained only a few populations of double drug-resistant *L. amazonensis* promastigotes which turned out to be unstable. The fact that those populations did not sustain sub-cultures precluded further analyses. Clearly, not all species or strains of *Leishmania* are equal in terms of capacity to generate mating-competent forms *in vitro*. Hence, whereas Louradour and colleagues were successful in recovering hybrids from *L. tropica* axenic co-cultures, no hybrids were obtained when both parental strains were *L. major* (Louradour et al., 2020). In the case of *L. amazonensis*, it is possible that strains other than the one we used are more efficient in generating mating-competent forms in axenic cultures. Future studies will be aimed at investigating this important issue.

It is well established that hybrid formation among *Leishmania* promastigotes takes place in the sand fly (Akopyants et al., 2009; Sadlova et al., 2011; Inbar et al., 2013; Calvo-Alvarez et al., 2014; Romano et al., 2014; Inbar et al., 2019; Louradour et al., 2020). Failure to detect genetic exchange in the mammalian host suggests that amastigotes do not generate mating competent forms or that they are less prone to recombination. It is also possible that the phagolysosomal environment is not as conducive to genetic exchange as the sand fly midgut. However, it was reported previously that amastigotes undergo nuclear fusion within infected macrophages suggesting that genetic exchange may indeed be possible within infected hosts (Kreutzer et al., 1994). Alternatively, the fact that the *Leishmania* species used so far to study genetic exchange replicate within individual parasitophorous vacuoles (*L. major*, *L. tropica*, *L. donovani*, *L. infantum*) may have limited the probabilities of genetic exchange among amastigotes. With this in mind, we hypothesized that replication within a communal vacuole may provide amastigotes with conditions propitious to genetic exchange, as reported for *Chlamydia* (Jeffrey et al., 2013). The recovery of double-drug resistant promastigote populations from macrophages co-infected with *L. amazonensis LPG2/LPG2::ΔHYG* + *L. amazonensis +/SSU::NEO-GFP* and the detection of both the *HYG* and *NEO* genes in these cultures suggest that intraclonal genetic exchange may occur within communal parasitophorous vacuoles. However, the inability to grow these *L. amazonensis* double drug-resistant populations over several passages and to clone double drug-resistant parasites precluded further characterization of these progeny and thus determine whether or not these parasites were genuine hybrids. Previous studies revealed that not all hybrid progeny is as viable as their parental counterparts. Hence, Sadlova et al. observed *L. donovani* hybrids in infected sand flies, but all of their attempts to grow them in culture have failed. This led to the conclusion that although *L. donovani* parasites are able to exchange genetic information, the hybrids produced were not viable (Sadlova et al., 2011). Finally, there was also a report which explored the possibility of genetic exchange between *L. major* and *L. turanica* in infected sand flies; however, it was reported that such events do not take place between these parasite species (Chajbullinova et al., 2012).

As described for *L. major* (Akopyants et al., 2009), we were unable to recover double drug-resistant parasites from mice co-infected with *L. amazonensis* and *L. mexicana*. However, based on our results with *in vitro* infections, we cannot rule out that genetic exchange do not take place in mammalian hosts infected with those species/strains. An important factor to consider is the number of *in vivo* infections we performed. Indeed, studies on genetic exchange done in the insect vector required hundreds of sand flies to be infected. Hence, Akopyants et al. used 102 sand flies to study genetic exchange between *L. major* parasites, Sadlova et al. infected 121 sandflies to study this phenomenon for *L. donovani*, whereas Romano et al. used 446 sandflies to study these events among strains of *L. infantum* (Akopyants et al., 2009; Sadlova et al., 2011; Romano et al., 2014). Another important factor to take into consideration is the ability of the *L. amazonensis* and *L. mexicana* strains we used in our study to generate mating competent forms, as evidenced in the study of Louradour and colleagues using *L. tropica* and *L. major* (Louradour et al., 2020).

In summary, we provide evidence of possible intraclonal genetic exchange among *L. amazonensis* parasites in axenic cultures and within mammalian host cells. However, the double drug-resistant parasites obtained in our studies were unstable and could not be further characterized. Future studies will be required to identify strains of *L. amazonensis* and *L. mexicana* with higher capacity to generate mating competent forms and use these strains for studies in macrophages and in mice on a larger scale.

## Data Availability Statement

The raw data supporting the conclusions of this article will be made available by the authors, without undue reservation.

## Ethics Statement

The animal study was reviewed and approved by Comité Institutionel de Protection des Animaux of the INRS-Centre Armand-Frappier Santé Biotechnologie.

## Author Contributions

RT and AD conceived and designed the study, contributed to the data analysis, and drafted and revised the manuscript. RT performed the experiments. RT and AD wrote and revised the manuscript. All authors contributed to the article and approved the submitted version.

## Funding

This work was supported by the Canadian Institutes of Health Research (CIHR) grant PJT-156416 to AD. AD is the holder of the Canada Research Chair on the Biology of intracellular parasitism. The funders had no role in study design, data collection and interpretation, decision to publish, or preparation of the manuscript.

## Conflict of Interest

The authors declare that the research was conducted in the absence of any commercial or financial relationships that could be construed as a potential conflict of interest.
